# Supplementary material for: Factors Associated with Prolonged Antibiotic Therapy in Neonates with Suspected Early-Onset Sepsis
Source: Antibiotics (Basel). 2024 Apr 25;13(5):388. doi: 10.3390/antibiotics13050388 (PMC11117245; doi:10.3390/antibiotics13050388)
Supplement: Supplementary file 1 [file antibiotics-13-00388-s001.zip › antibiotics-2962981-supplementary.pdf]

SUPPLEMENTAL MATERIAL

Supplement 1. Maternal Risk Factors & Neonatal Clinical Symptoms
Supplement 2. Flowchart Dutch Guideline
Supplement 3. Reasons to Continue Antibiotic Therapy
References

Supplement 1. Maternal Risk Factors & Neonatal Clinical Symptoms

Table S1. Maternal Risk Factors & Neonatal Clinical Symptoms

Shown are the maternal risk factors and neonatal clinical signs according to the Dutch guideline,[1] which is an adaptation of the NICE guideline.[2] This table was published earlier as part of a randomised controlled trial protocol.[3]

Maternal risk factors	Neonatal risk factors
**Red flags**	
Parenteral antibiotic treatment given to the woman for confirmed or suspected invasive bacterial infection (such as septicemia) at any time during labor, or in the 24-hour periods before and after the birth	Respiratory distress starting more than 4 hours after birth
Suspected or confirmed infection in another neonate in case of a multiple pregnancy	Neonatal epileptic seizures
	Need for mechanical ventilation in a term neonate
	Signs of shock
**Non-red flags**	
Invasive group B streptococcal infection in a previous neonate	Altered behavior, -responsiveness or -muscle tone
Maternal group B streptococcal colonization, bacteriuria or infection in the current pregnancy	Feeding difficulties (feed refusal, gastric retention, vomiting, distended abdomen)
Suspected or confirmed rupture of membranes without contractions for more than 24 hours in a term birth	Apnea and bradycardia
Preterm birth following spontaneous labor (before 37 weeks' gestation)	Signs of respiratory distress (tachypnea, moaning, retractions, nasal flaring)
Suspected or confirmed rupture of membranes for more than 18 hours in a preterm birth	Hypoxia (for example, central cyanosis or reduced oxygen saturation level)
Intrapartum fever higher than 38°C or suspected or confirmed chorioamnionitis	Neonatal encephalopathy
	Need for cardio-pulmonary resuscitation
	Need for mechanical ventilation in a preterm neonate
	Persistent pulmonary hypertension
	Temperature abnormality (lower than 36°C or higher than 38°C) unexplained by environmental factors
	Local signs of infection (for example, affecting the skin or eyes)

Abbreviation: NICE, National Institute for Health and Care Excellence.

Supplement 2. Flowchart Dutch Guideline

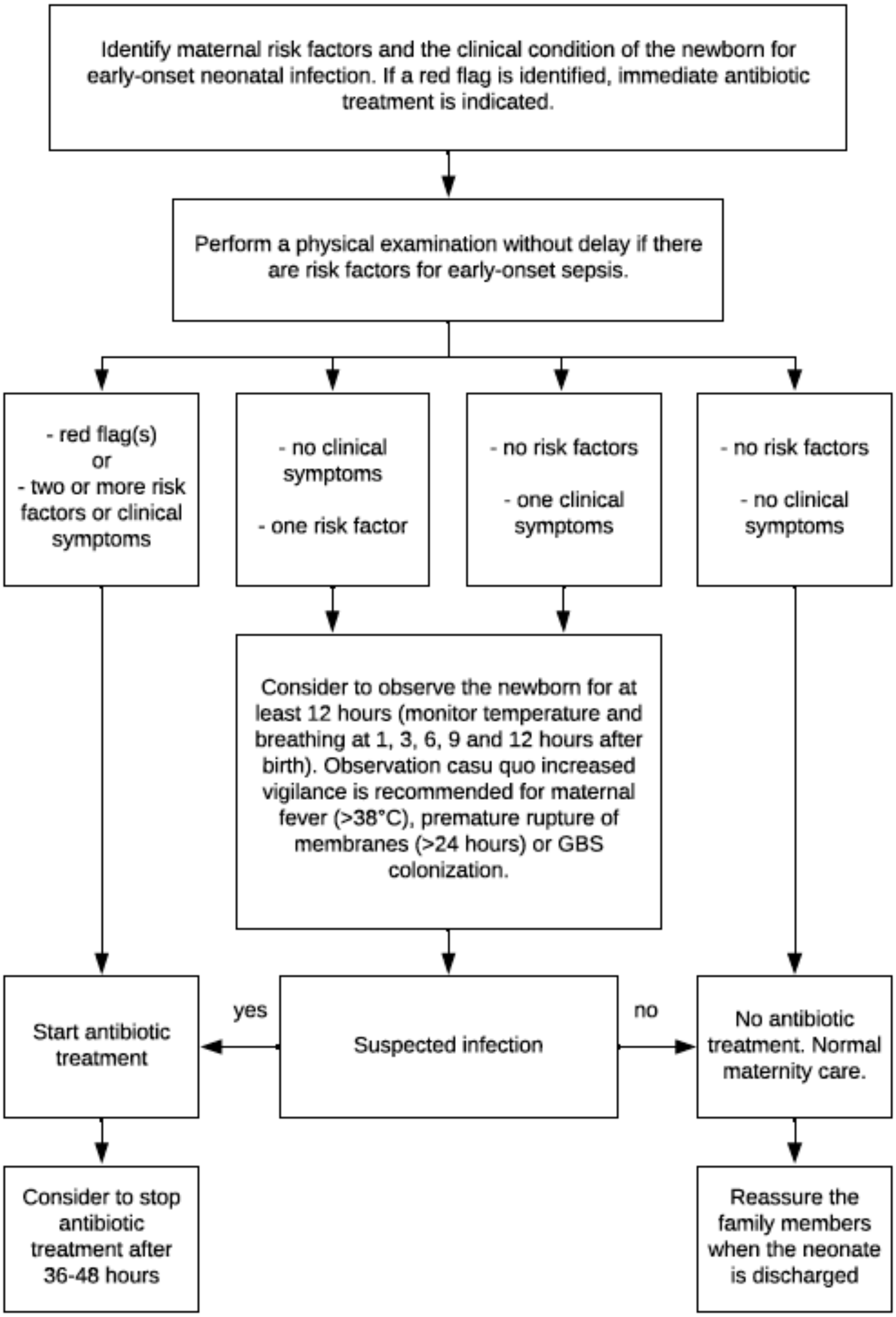

Figure S1. Flowchart Dutch Guideline

Shown is the adaptation of the algorithm used in the Dutch guideline to provide guidance for antibiotic treatment prescription according to the number of present maternal risk factors and/or neonatal clinical symptoms, categorised in red flags and non-red flags.[1] This figure was previously published as supplemental material of a multicentre study.[4]

Supplement 3. Reasons to Continue Antibiotic Therapy

Table S2. Reasons to continue antibiotic therapy in neonates with a negative blood culture.

	**Prolonged AB (n=47)**
**Neonatal illness at start** (n (%))	4 (8.5)
**Apgar score** (n (%))	1 (2.1)
**Maternal risk factors** (n (%))	3 (6.4)
**Heart rate** (n (%))	1 (2.1)
**Respiratory rate** (n (%))	3 (6.4)
**Temperature** (n (%))	3 (6.4)
**Clinical appearance** (n (%))	18 (38.3)
**Skin colour** (n (%))	5 (10.6)
**Neurological state** (n (%))	2 (4.3)
**CRP level** (n (%))	17 (36.2)
**WBC count** (n (%))	1 (2.1)
**Apnoea/Bradycardia** (n (%))	1 (2.1)
**Feeding difficulties** (n (%))**s**	1 (2.1)
**Not noted** (n (%))	12 (25.5)
**Advice NICU** (n (%))	3 (6.4)
**False negative blood culture due to maternal antibiotic use** (n (%))	1 (2.1)
**X-ray** (n (%))	2 (4.3)

Abbreviations: CRP, C-reactive protein; NICU, neonatal intensive care unit; WBC, white blood cell.

References

1. NVOG (Nederlandse Vereniging voor Obstetrie en Gynaecologie), NVK (Nederlandse Vereniging voor Kindergeneeskunde). Preventie en behandeling van early-onset neonatale infecties (Adaptatie van de NICE-richtlijn). 2017;(april):1-94.
2. National Institute for Health and Clinical Excellence. Neonatal infection (early onset): Antibiotics for prevention and treatment. Clinical Guideline. Published 2012. https://www.nice.org.uk/guidance/cg149/resources/neonatal-infection-early-onset-antibiotics-for-prevention-and-treatment-35109579233221
3. van der Weijden BM, van der Weide MC, Plötz FB, Achten NB. Evaluating safety and effectiveness of the early-onset sepsis calculator to reduce antibiotic exposure in Dutch at-risk newborns: a protocol for a cluster randomised controlled trial. *BMJ Open*. 2023;13(2):1-8. doi:10.1136/bmjopen-2022-069253
4. van der Weijden BM, Achten NB, Bekhof J, Evers EE, Berk M, Kamps AWA, et al. Multicentre study found that adherence to national antibiotic recommendations for neonatal early-onset sepsis was low. *Acta Paediatr Int J Paediatr*. 2021;110(3):791-798. doi:10.1111/apa.15488
